# An Unusual Case of Madelung's Disease with Multiple Atypical Fractures

**DOI:** 10.1155/2012/180506

**Published:** 2012-12-31

**Authors:** Abdurrahman Tufan, Ridvan Mercan, Arif Kaya, Mehmet Engin Tezcan, Berivan Bitik, Mehmet Akif Ozturk, Seminur Haznedaroğlu, Berna Goker

**Affiliations:** Division of Rheumatology, Department of Internal Medicine, Faculty of Medicine, Gazi University, Ic Hastalıkları ABD, Romatoloji BD, Besevler, 06500 Ankara, Turkey

## Abstract

Madelung's disease is a rare acquired disorder of fat metabolism characterized by multiple symmetric lipomas with typical distribution mainly around the upper trunk, neck, and shoulders. The condition is strongly associated with chronic alcohol use and has various systemic manifestations like polyneuropathy, muscle weakness, and small bone fractures. Herein, we report a 56-year-old male patient with Madelung's disease and multiple fractures and discuss possible underlying factors leading to multiple fractures.

## 1. Introduction

Madelung's disease (benign multiple symmetric lipomatosis, MSL) is a rare condition characterized by the multiple, nonencapsulated, infiltrative lipomas located symmetrically on the trunk, neck, and proximal parts of the limbs [1]. Although etiology of the disease is unknown, there is history of heavy alcohol drinking in 60–90% of cases [1, 2]. The disease occurs more commonly in middle-aged men. Other features of MSL include polyneuropathy, proximal muscle weakness, and fractures of small bones [1, 3, 4]. Upper airway obstruction due to compression by lipomas and sudden heart death are the most frequent causes of fatality [5]. There is no effective treatment, though alcohol abstinence and surgical removal are current approaches [2]. Herein, we present a case of MSL with multiple fractures located at atypical locations. 

## 2. Case Report

**Figure 1:**
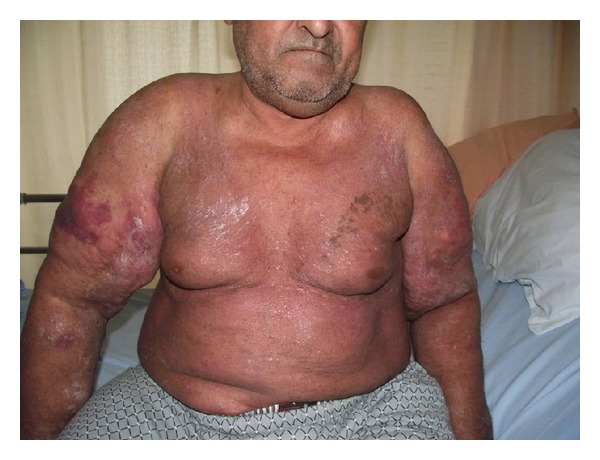
Frontal plane picture showing symmetric lipomatosis lesions at pectoral area and upper arms.

A 56-year-old male retired as coal worker admitted for progressive proximal muscle weakness for 1-year duration. His past medical history was significant for heavy smoking with 50 pack-years and heavy drinking for 20 years. He had had minor traumatic left proximal tibia and clavicle fractures 7 and 1 years ago, respectively. He had psoriasis vulgaris for forty years and had been using topical steroids for his psoriatic lesions for the last 6 months prior to admission. He had been bedridden for the last 6 months due to muscle weakness. He denied any major trauma or falls. His physical examination revealed abdominal obesity, coarse breath sounds, and extensive erythematosus exfoliative skin lesions on his trunk. There were also multiple lipomatous lesions distributed on the upper arms, pectoral area, and neck (Figure 1). His proximal muscle strength was of grade 3-4/5, while distal muscles had normal strength. Laboratory tests showed a hemoglobin level of 12.1 g/dL with mild macrocytosis, 2- and 4-fold increases in transaminases and alkaline phosphatase, respectively. Serum vitamin B12 (305 pg/mL), folic acid, creatine kinase level, and electromyography were normal. Plain radiographies of the chest, pelvis, and knee demonstrated old fractures located at the clavicles, left proximal tibia, pubic ramus, and right femoral head. The computed tomography of the thorax revealed multiple lipoma lesions—various in size—over the trunk and arms, bilateral costal and scapular healed fractures, as well as fractures of the thoracal and lomber vertebral transverse and spinous processes. His serum calcium, phosphorus, and magnesium concentrations and 24-hour urinary calcium excretion were within normal limits. However, serum 25-hydroxy vitamin D level was very low (5.4 ng/mL, normal range: 10–55) and intact parathyroid hormone level significantly elevated (311 pg/mL, normal range 12–72). Bone mineral densitometry showed an apparent osteoporosis with total femoral and lomber *T* scores of −2.8 and −4.3, respectively. His serum basal cortisol and adrenocorticotropic hormone levels were consistent with iatrogenic hypercortisolism (basal cortisol: 2.3, normal range 6–30 mg/dL, ACTH: 8.8, normal range <60 pg/mL). Liver ultrasonography demonstrated irregular liver contours and caudate lobe hypertrophy, consistent with chronic liver disease. Esophageal varices were observed via esophagogastroduodenoscopy. Viral hepatitis and autoimmune liver serologic markers were all negative. Skin biopsy obtained from abdomen was reported as psoriasis.

Patient's nutritional requirements were evaluated, and requirements of trace elements and vitamins, including vitamin D, were replaced parenterally. He was advised to quit alcohol and received medical support for this. He was provided a physiotherapy program. With supportive therapy, he was able to mobilize, initially with assistance and then independently within two months. His long-term medical therapy included nutritional support, teriparatide, vitamin D, and calcium supplementation. Low-dose oral steroid and cyclosporine were initiated for generalized erythematous psoriasis. He was doing well at his follow-up visit at 4 months after discharge. His psoriatic lesions were in remission; however symmetric lipomatosis remained unchanged.

## 3. Discussion

MSL is a metabolic fat disorder characterized by the growth of fatty masses around the face, neck, upper trunk, and proximal parts of limbs with a symmetric distribution [1]. MSL affects mainly white males with a male/female ratio of 15 : 1 [6]. The condition has been regarded as very rare. Its prevalence is highest in the Mediterranean area with an estimated incidence of 1/25,000 in Italy [7]. MSL predominantly manifests itself in third to fifth decades of life. The disease usually has biphasic course, an initial rapid growth that is followed by a slow progressive phase. Currently spontaneous regression has not been reported. 

The exact cause of this disease is unknown, but there is a strong association with heavy alcohol intake [5]. The use of protease inhibitors is another well-defined risk factor [8]. Alcohol causes reduction in number, and activity of beta adrenergic receptors therefore promote lipogenesis which may explain its pathogenetic role. There are familial MSL cases in the literature, and mitochondrial DNA mutations are observed in most subjects, suggesting a genetic predisposition [9]. 

Apart from typical disfiguring lipomatosis, MSL has other systemic manifestations which are part of this syndrome. The most common manifestations are related to neuropathies involving motor, sensory, and autonomic nerves, with a reported incidence as high as 85% [1, 3, 4]. Neurologic symptoms are weakness and areas of anesthesia and paresthesias, as well as autonomic nervous system manifestations such as flushing and sweating, fluctuations in blood pressure and heart rate, adult onset asthma, glucose intolerance, gastrointestinal problems, chronic diarrhea, and foot problems, such as ulcers on the plantar surface of the foot or spontaneous fractures of small bones.

Our case is a middle-aged man with a long standing history of heavy ethanol use. The patient's initial presentation was mostly related to the neurological manifestations. He presented with paresthesia, apparent proximal muscle weakness and palpitations, diarrhea, and flushing. He had no complaints related to the lipomatosis, probably due to their noncritical locations. The most striking feature of the patient was multiple atypical spontaneous fractures. He denied any previous major trauma or falls, as well as symptoms related to the fractures. Anesthesia secondary to neuropathy or analgesic effect of ethanol use by itself might have contributed to the lack of fracture symptoms. Such widespread fractures might have been facilitated by the long-term use of topical potent corticosteroid and nutritional deficiencies secondary to chronic alcoholism. He had significant clinical and functional improvement with nutritional support and alcohol abstinence, as well as physiotherapy. However, as reported in the literature, no regression was observed in the lipomatous lesions after alcohol abstinence and medical therapy.
